# Aortic calcification accelerates cardiac dysfunction via inducing apoptosis of cardiomyocytes

**DOI:** 10.7150/ijms.90324

**Published:** 2024-01-01

**Authors:** Nannan Hao, Hui Yong, Feifei Zhang, Chang Liu, Yulu Qiu, Yumeng Shi, Chunjian Li, Fang Wang

**Affiliations:** 1 Department of Cardiology, the First Affiliated Hospital of Nanjing Medical University, China.; 2 Department of Rheumatology, the First Affiliated Hospital of Nanjing Medical University, China.

**Keywords:** vascular calcification, cardiac dysfunction, artery stiffness, apoptosis, vitamin D_3_

## Abstract

Vascular calcification (VC) is a known predictor of cardiovascular events in patients with atherosclerosis and chronic renal disease. However, the exact relationship between VC and cardiovascular mortality remains unclear. Herein, we investigated the underlying mechanisms between VC progression, arterial stiffness, and cardiac dysfunction. C57BL/6 mice were administered intraperitoneally vitamin D_3_ (VD_3_) at a dosage of 35×10^4^ IU/day for 14 days. At day 42, VC extent, artery elasticity, carotid artery blood flow, aorta pulse propagation velocity, cardiac function, and pathological changes were evaluated. Heart apoptosis was detected using TUNEL and immunohistochemistry staining. In *vitro*, rat cardiomyocytes H9C2 were exposed to media from calcified rat vascular smooth muscle cells (VSMCs) cultured in calcification medium, and then H9C2 apoptosis and gene expression related to cardiac function were assessed. VD_3_-treated mice displayed a significant aortic calcification, increased pulse propagation velocity of aortae, and reduced cardiac function. Aortae showed increased calcification and elastolysis, with increased heart apoptosis. Hearts demonstrated higher levels of ANP, BNP, MMP2, and lower levels of bcl2/bax. Moreover, calcified rat VSMC media induced H9C2 apoptosis and upregulated genes expression linked to cardiac dysfunction. Our data provide evidence that VC accelerates cardiac dysfunction, partially by inducing cardiomyocytes apoptosis.

## Introduction

Arterial wall stiffness is well-documented as a strong independent predictor of cardiovascular morbidity and all-cause mortality 1, 2. Vascular calcification (VC) has been suggested to be a mechanism underlying development of arterial stiffness of large arteries 3, 4. VC is defined as the extracellular deposition of pathological mineral, in a form of calcium-phosphate complexes, in the arterial wall 5, 6. Traditionally, VC is classified into two forms depending on the mineral deposition within intima or media of the arterial wall. New research data from molecular pathogenesis illustrate that intimal and medial calcification are distinct in their morphology and may represent different pathological conditions 7, 8. However, both forms of VC often coincide and overlap in one disease.

Accumulating evidence support a view that VC is not a passive but actively cell-regulated biologic process, and vascular smooth vascular cells (VSMCs) are suggested to be the main cell type responsible for VC 5, 9, 10. Dysfunctional VSMCs in the process of intima or media calcification often coincide and occur in patients with cardiovascular diseases, because their triggering factors, such as aging, diabetes mellitus, and uraemia, frequently overlap 11.

The association of VC with cardiovascular diseases has been long suggested. Importantly, VC, as well as arterial stiffness which represents the functional disturbance of VC, have been reported to strongly and reciprocally correlate in many clinical studies 5, 12, 13. Theoretically, increased calcium deposition in the large arteries causes arterial stiffness and further results in left ventricular structural and functional changes 12, 13. Clinically, it has been reported heavy aortic calcification causes left ventricular dysfunction in coronary artery disease (CAD), hemodialysis and chronic kidney disease (CKD) patients 12. There may be a common pathophysiological pathway linking aortic calcification and cardiac dysfunction in patient who had a high aortic calcium burden whatever deposition in the intimal and/or medial vessel layer 12. Indeed, worsened cardiac dysfunction has also been noted in several calcified mice, such as vitamin D_3_ (VD_3_) plus warfarin-treated 5/6 nephrectomy C57BL/6 mice 14, VD_3_ -treated ob/ob mice 15, and LDLR^-/-^ mice fed high fat diets (HFD) 16. However, the precise mechanisms of VC being responsible for cardiac dysfunction have not been completely clarified. Therefore, understanding the factors and mechanisms driving these processes will provide novel therapeutic targets for its prevention.

To examine the mechanism of VC promoting cardiac dysfunction, we used a well-defined VD_3_ -induced calcified mice to analyze the changes of aortic and cardiac function parameters. Moreover, we addressed the potential mechanism of the association by which calcification may portend poor cardiovascular function by increased cardiomyocytes apoptosis, oxidative stress and MMP expression.

## Materials and methods

### Reagents

Vitamin D_3_ was from Sigma (Saint Louis, MO, USA). PrimeScript®RT Master Mix was from TakaRa (Dalian, China) and SYBR Green PCR Master Mix was from Applied Biosystems (Carlsbad, CA, USA). TUNEL, Weigert and Picro Sirius Red (PSR) staining solution from Beyotime (Shanghai, China), Alizarin Red S and von kossa staining solution from Solarbio (Beijing, China). Primary antibody bcl-2, bax and β-actin from Proteintech (Rosemont, IL, USA). Dulbecco's modified Eagle's medium (DMEM) and other culture reagents were from GIBCO (Carlsbad, CA, USA).

### Vitamin D_3_-induced vascular calcification in mice

Male C57BL/6 mice (8 weeks old) were purchased from GemPharmatech (Nanjing, China). The animal experiment was approved by the animal ethics and welfare committee of the Nanjing Medical University (Nanjing, China). The induction of VC in mice with VD_3_ was performed as we previously described 17. Briefly, mice (n = 10) received intraperitoneal injection with vitamin D_3_ (350000 IU/kg per day) dissolved in olive oil for 14 consecutive days, and the same volume olive oil injection was used as a control. The body weight of mice was recorded every 3 days during the animal experiment. At day 28 and 42, mouse aortic stiffening and cardiac function were determined under isoflurane anesthesia, respectively. Finally, at day 42, after detection of blood flow of mouse carotid artery in anesthesia, then mouse blood and tissues (aortas, hearts, livers and kidneys) were collected, and the latter were kept in 4% paraformaldehyde solution or snap-frozen in liquid nitrogen.

### Serum assay of calcium content and alkaline phosphatase (ALP) activity

Mouse serum levels of calcium were measured on an automatic biochemistry analyzer (Rayto Chemray-800, China). ALP activity was analyzed by a *p*-nitrophenyl phosphate (PNPP) method using an ALP Activity Assay kit (Elabscience, China) as per the manufacture's protocol. Briefly, mouse serum (5 μL) was incubated with ALP reagent (50 µL) at 37 ℃ for 15 min. After adding the detecting reagent, the absorbance was measured at 520 nm and compared to a standard curve to calculate the ALP activity (U/100 mL).

### Measurement of Pulse Propagation Velocity (PPV) in the abdominal aorta

At day 28 and 42, PPV was measured in the abdominal aorta of mouse during isoflurane anesthesia (1%-1.5%) by ultrasound (Vevo 3100, MX400 transducer) using EKV-image acquisition. Analysis was performed with the Vevo-Vasc software (VisualSonics, Inc., Toronto, Canada).

### Laser speckle contrast imaging (LSCI) in the carotid artery

The left carotid artery of mouse was isolated carefully and carotid artery blood flow was measured using the Moor FLPI Full-field Laser Perfusion Imager (MoorFLPI, Axminister, UK) with isoflurane anesthesia at day 42. The laser camera was positioned at 18-20 cm above the carotid artery, and blood flow values (Flux) were obtained within 5 min after local blood flow was stable. The blood flow images were color coded as described by Li et al. 18.

### M-mode echocardiography

Transthoracic echocardiograms were performed on mouse at day 28 and 42 using a Vevo 2100 ultrasound system (VisualSonics Inc., Toronto, Canada) to determine left ventricular (LV) function as we previously described 19. The heart rate, left ventricular mass (LV mass), end-systolic LV anterior wall thickness (LVAWs), end-diastolic LV anterior wall thickness (LVAWd), end-systolic LV posterior wall thickness (LVPWs) and end-diastolic LV posterior wall thickness (LVPWd) were measured. Left ventricular ejection fraction (LVEF), fractional shortening (FS), cardiac output and cardiac index (CI) were calculated. All measures were averaged over three consecutive cardiac cycles.

### Histological and immunohistochemical stainings

Mouse aorta, heart and kidney were fixed in 4% paraformaldehyde (PFA) and embedded in paraffin. The sections (5 - 7 µm) were used for different stainings. The hematoxylin and eosin (H&E) staining is to examine histopathological changes, Weigert staining for elastic and collagen fibers in aorta, as well as masson and PSR staining for heart fibrosis, according to the manufacturer's protocol.

ARS staining was used for calcification determination. The ARS staining for whole aortas and paraffin-embedded kidney, as well as vascular smooth muscles cells (VSMCs) and optical density 20 quantitation were adopted as those used in our previous study 17. Calcified areas are shown as red staining.

As to immunohistochemical staining to detect bcl-2 and bax expression in mouse heart, heart sections were deparaffinized and then subjected to heat antigen retrieval in 0.01 mM sodium citrate buffer (pH 6.0) and inactivation of endogenous peroxidase with 3% H_2_O_2_. After blocking with 5% bovine serum albumin (BSA) to reduce non-specific background staining, sections were incubated with primary antibody bcl2 and bax overnight at 4 ℃, and next incubated with a secondary antibody for 1 h at room temperature. Finally, the reactions were developed using a 3,3-N-diaminobenzidine tertrahydrochloride substrate kit (Beyotime, Shanghai, China).

All the slides were scanned at ×20 via an Aperio ScanScope XT slide scanner, and the digital image was observed with ImageScope software (Leica Biosystems, Nussloch, Germany).

### Cell culture and treatment

The rat cardiomyocyte H9C2 (Cell Resource Center of Chinese Academy of Sciences, Shanghai, China) was cultured in DMEM supplemented with 10% fetal bovine serum (FBS), 100 U/ml of penicillin, and 100 mg/ml of streptomycin at 37 ℃ in a 5% CO_2_ humidified atmosphere.

Primary rat aortic smooth muscle cells (RVSMCs) were isolated from adult male Sprague Dawley rats (Jiangsu Laboratory Animal Center, Nanjing, China) as we previously described 17. Briefly, thoracic aortas were cut into 1 - 2 mm sections in sterile and cultured in DMEM/F12 medium supplemented with 20% FBS, 100 U/mL penicillin, and 100 μg/mL streptomycin. After 5 - 7 days, migrated RVSMCs were sprouted from the tissue sections, and 3 to 8 passages were used for subsequent experiments.

To induce the calcification of VSMCs in vitro, RVSMCs were cultured in calcification medium containing 1.5 mM calcium, and 2.0 mM phosphate for a further 2 to 3 days incubation as described previously 17, 21. And the supernatants were collected as conditional media for further study.

To study the effect of calcified VSMC on H9C2 function, we provoked H9C2 cells with various dilution of conditional media from calcified RVSMC for 48 h. Media from RVSMCs only treated with DMEM/F12 medium was used as controls. After incubation, H9C2 cells were collected for gene expression analysis and TUNEL staining. Three independent experiments were carried out in triplicates each group.

### Terminal deoxyribonucleotidyl transferase (TdT)-labeling (TUNEL) assay

Mouse heart sections (5 - 7 µm) in paraffin and H9C2 in 4% PFA were stained using TUNEL assay to determine the apoptosis of cardiomyocytes, as described by the manufacture's protocol, respectively. TUNEL-positive cells were imaged by a Zeiss fluorescence microscope (Zeiss, Oberkochen, Germany) and quantified with Image J software. Three to five random fields in each section were counted (3 sections/ specimen from 5 mice per group) over a microscopic field of 20×, and expressed as the ratio of TUNEL-positive nuclei (%) 22. Similarly, 5 - 7 random fields in each well were counted in H9C2 cells (3 wells/group).

### Real-time quantitative polymerase chain reaction (RT-qPCR)

Mouse hearts and H9C2 cells were treated with 1 ml of TRIzol reagent (Vazyme, Nanjing, China). Total RNA was extracted and then reverse-transcribed using a reverse-transcription kit (Takara, Dalian, China) following the manufacturer's instructions. RT-qPCR was performed using the QuantStudio™ 5 Real-Time PCR System (Applied Biosystems, UK) with the Power SYBR Green PCR Master Mix (Applied Biosystems, UK). The primers for β-actin, atrial natriuretic peptide (ANP), brain natriuretic peptide (BNP), matrix metalloproteinase 2 (MMP2), matrix metalloproteinase 9 (MMP9), tumor necrosis factor-α (TNF-α), tumor growth factor β (TGF-β), interleukin 6 (IL-6), collagen type Ⅰ alpha 1 (Col1a1) and collagen type Ⅲ alpha 1 (Col3a1) are shown in Supplementary Table 1. The relative gene expression was normalized to that of the house keeping gene β-actin using the 2^-ΔΔCt^ method.

### Statistical analysis

Data were expressed as mean ± standard error (SEM). Differences between the 2 groups were compared by a 2-tailed unpaired Student t test or Mann Whitney U test. Multiple-sample comparisons were analyzed by ordinary 1-way ANOVA with Tukey multiple comparisons test. All statistical analyses and figures were performed using GraphPad Prism 9.0 software (GraphPad software Inc., CA, USA). P<0.05 was considered statistically significant.

## Results

### Evaluation of VD_3_-induced VC mice model

**Figure 1:**
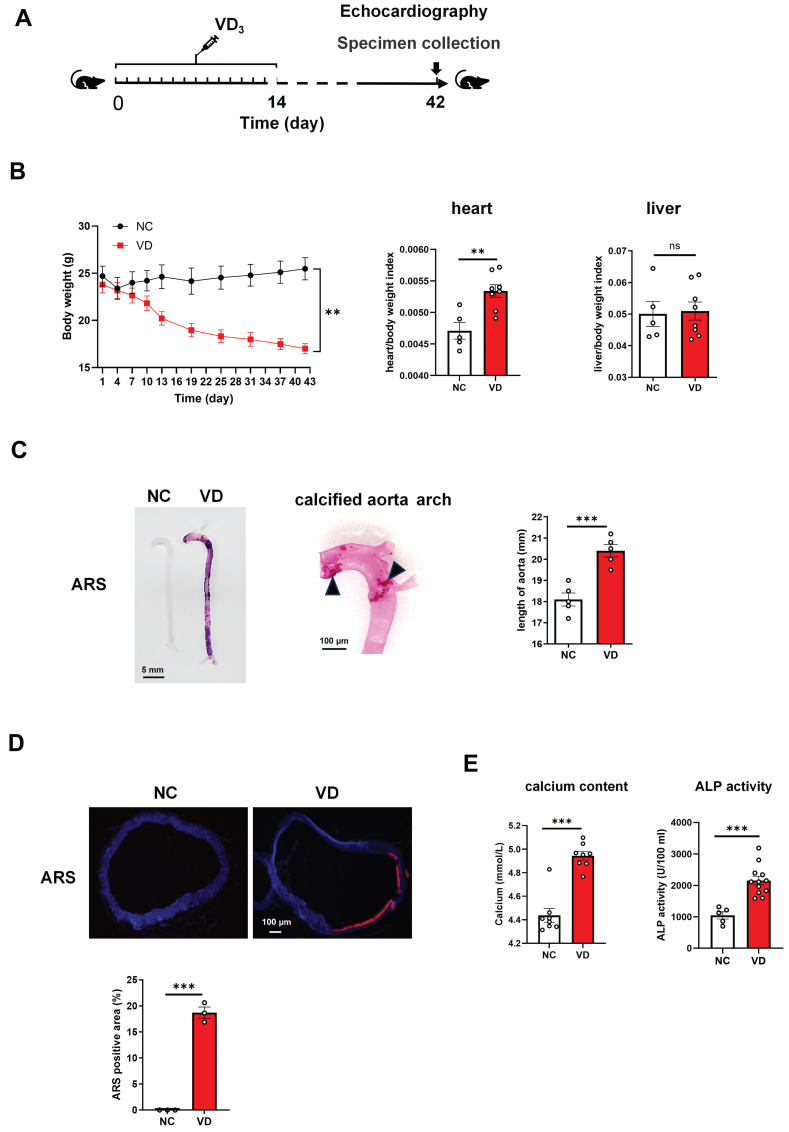
** Vitamin D_3_ (VD_3_) administration triggers the development of vascular calcification in C57BL/6 mice. (A)** Experimental timeline. C57BL/6 mice (n = 10 per group) were intraperitoneally injected with VD_3_ (350,000 IU/kg/day) for 14 days. At day 42, mouse abdominal ultrasonic and echocardiography were performed respectively, and at day 42 blood and tissue samples were collected. **(B)** Mouse body weight was monitored during the experiment, heart and liver weight was determined at the end of the experiment. **(C)** Representative ARS staining for whole aorta of mouse (left), a representative calcified aortic arch from a VD_3_-induced mice (middle), and the comparation of aorta length between controls and VD_3_-treated mice (right). The solid triangles showed marked calcification deposition.** (D)** Representative ARS images for aortic sections and quantification of the percentage of ARS positive areas showing calcification location (red). **(E)** The serum calcium levels and ALP activity in mice were measured at the end of the experiment. Data are shown as mean ± SEM. **P < 0.01, ***P < 0.001. ns: no significant, ARS: Alizarin Red S.

C57BL/6 mice received 350000 IU/kg per day of intraperitoneal VD_3_ for consecutive 14 days, and at day 28 and 42 their vascular and cardiac function were determined as well as samples were collected at day 42 (Figure 1A). In VD_3_-treated mice (VD group), body weight decreased around day 11 and kept this decreasing trend until the end of the experiment (Figure 1B, left). At the end of the animal experiment, all mouse heart, kidney and liver weighted, and the ratio of heart/body weight and kidney/body weight in VD group was significantly higher than normal controls (NC group), while liver/body weight showed no significant change (Figure 1B; Supplementary Figure 1; Supplementary Table 2).

Aortae from VD_3_-treated mouse showed strikingly increased VC detected by ARS staining showing both in the whole length of aortae and enlarged aorta arch at the macroscopic level (Figure 1C). Additionally, the length of aorta from aortic root to iliac artery from VD group showed statistically significant increase when compared with those from NC group (Figure 1C, right), which partially reflected the severity of arterial calcification.

In the cross-sectional aorta, ARS staining with dark field illumination analysis 23 depicted augmented VC characterized by red calcium deposition (Figure 1D, upper), and the percentage of calcification area in relation to the total vascular area in VD_3_-treated mice compared with untreated ones (Figure 1D, below).

Furthermore, the exaggerated VC response of VD treatment was accompanied by a statistically significant increase in serum calcium content and ALP activity versus non-treated ones (Figure 1E). Together, these results suggested that VC was generated in mouse aorta after VD_3_ stimulation in vivo, and this animal model was used in the subsequent studies.

### Impaired vascular stiffness and cardiac function in calcified mice

**Figure 2:**
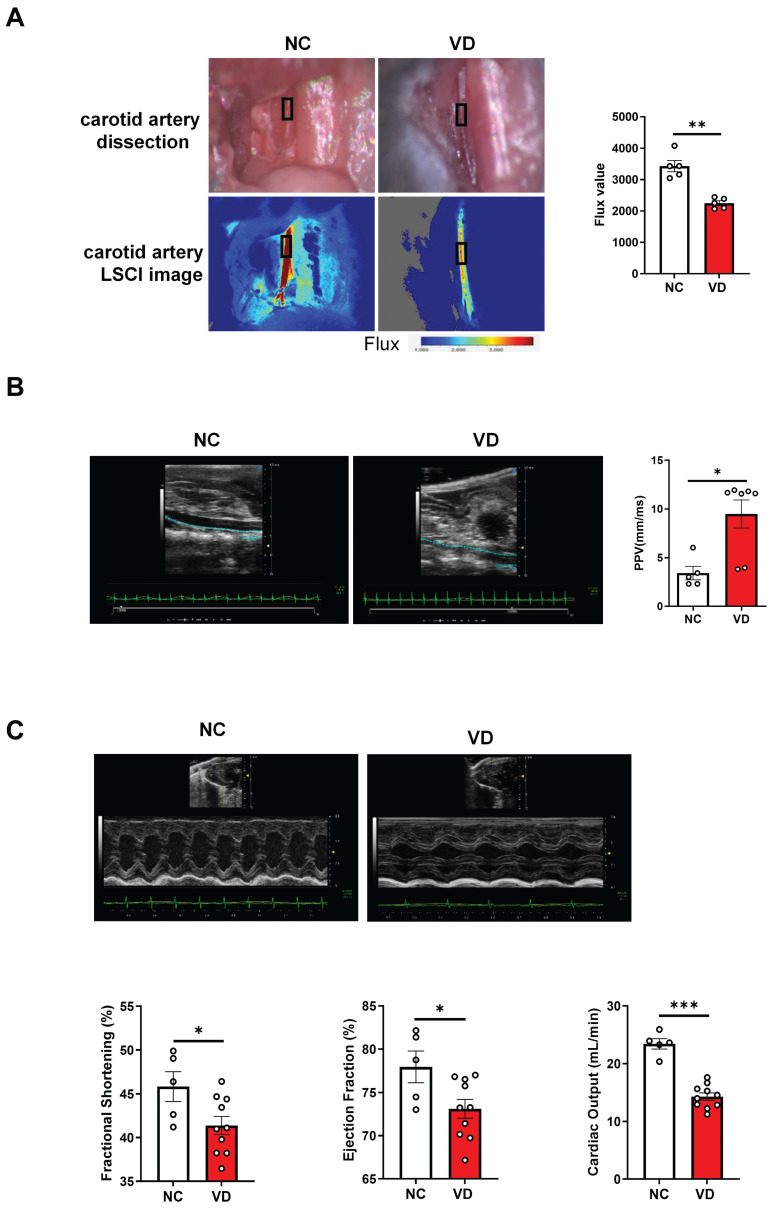
**Impaired blood flow, aorta pulse propagation velocity (PPV) and cardiac function in VD_3_-induced VC mice. (A)** Representative and quantification of laser speckle contrast imaging (LSCI) of the blood flow of carotid artery (n = 5 per group).** (B)** Representative and quantification of PPV of aorta with abdominal ultrasonic for mice (NC = 5; VD = 5 - 7). **(C)** Representative M-mode images of mouse hearts and quantification of ejection fraction (EF), fractional shortening (FS), and cardiac output (NC = 5; VD = 10). Data are shown as mean ± SEM. *P < 0.05, ***P < 0.001.

Next, taking advantage of functional imaging, we investigated the functional changes of the aorta and heart in VC mice. LSCI is a new non-scanning optical technique for observing blood flow, and now is widely used for imaging vascular structure and blood flow dynamics 24. The left carotid artery of mouse was dissected carefully under anesthesia (Figure 2A) at day 42 and its blood flow was examined using LSCI. As shown in Figure 2B, VD_3_ treatment led to significant decrease of blood flow compared to normal controls, suggesting reduced dilation of carotid artery in VC mice. Furthermore, aortic stiffness in vivo was measured in the abdominal aorta under isoflurane anesthesia by ultrasound at day 28 and 42, respectively, and we observed the aortic PPV after VD_3_ treatment in mice was significantly increased compared with normal controls at day 42 (Figure 2B), suggesting higher aortic stiffness in VC mice, but showed no difference between day 28 and 42 (data not shown). Finally, cardiac functions of VC mice were assessed using M-mode echocardiography day 28 and 42, respectively. As shown in Figure 2C and Supplementary Table 3, EF and FS as markers of cardiac dysfunction were significantly decreased in VC mice compared with normal ones at day 42. Cardiac output, LV mass, heart rate, LVAW, LVPW, stroke volume and CI were also significantly lower in VC mice than in normal ones. While all of the changes remained similar at day 28 (data not shown). Taken together, these imaging and ultrasonic findings indicate both aortic and cardiac dysfunctions were present at day 28 in this VC mice model.

### Pathological changes of aorta and heart in calcified mice

**Figure 3:**
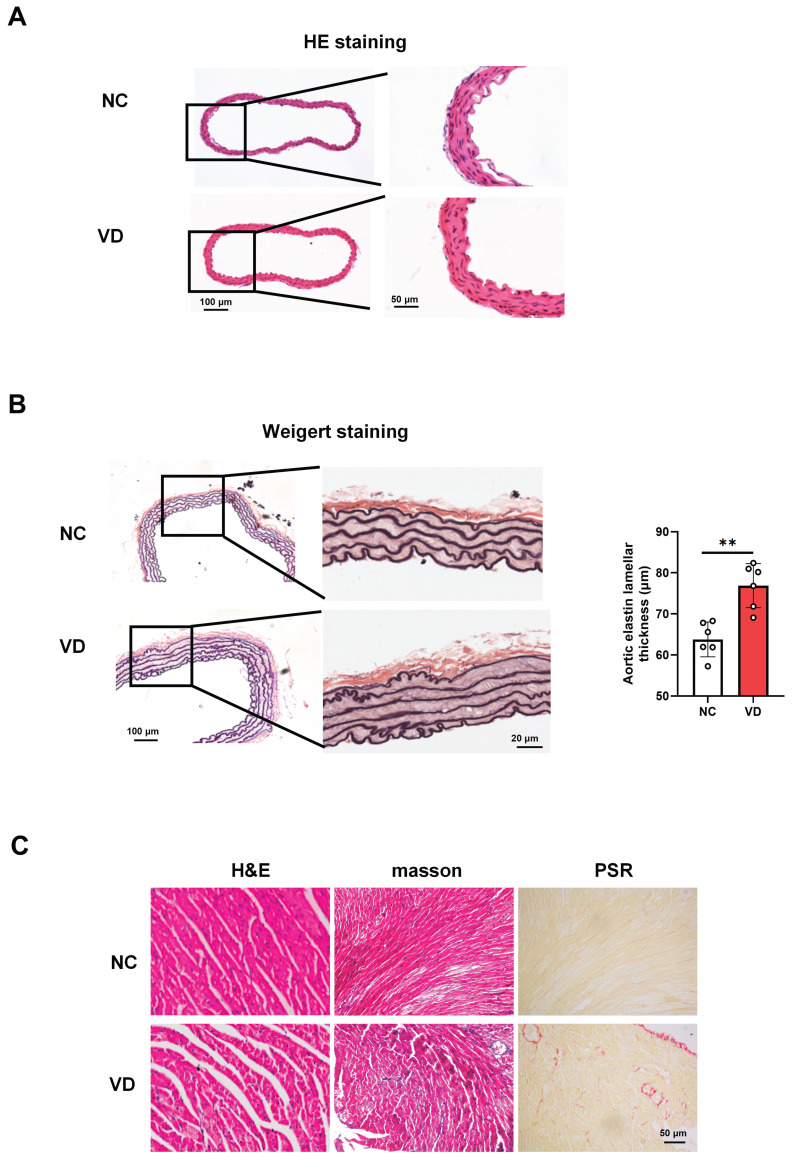
** Histopathological changes of aorta and heart in VD_3_-induced VC mice. (A)** Representative images of H&E staining for mouse aorta. **(B)** Representative images and quantification of Weigert staining for elastic fibers in mouse aorta. Data are shown as mean ± SEM (n = 5 - 6 per group). ***P < 0.001. **(C - D)** Representative images of histological staining for mouse hearts including H&E, masson and PSR (Picro Sirius Red) staining, respectively.

The presence of aberrant function both aorta and heart of VC mice strongly suggested that aorta and heart histology would be altered. A series of experiments explored the effect of VC on pathological changes in aorta and heart of mice (Figure 3). As to mouse aorta, there is no observed differences between VD_3_-treated mice and normal ones with H&E staining (Figure 3A), while broken elastic fibers and elastin thickness were obviously shown in VC mice with Weigert staining, indicating the increased vascular stiffness in calcified vessel (Figure 3B). In terms of the VC mouse heart, as illustrated in Figure 3C, cross striation was obscure or disappeared, and slightly increased inflammatory cell infiltration (H&E staining) and fibrosis (masson and PSR staining) were also shown in VC mice. These pathological results suggest calcified mice exhibit cardiovascular abnormalities which might account for the decreased heart performance.

### Aberrant expression of cardiac genes and cardiomyocytes dysfunction in hearts from calcified mice

**Figure 4:**
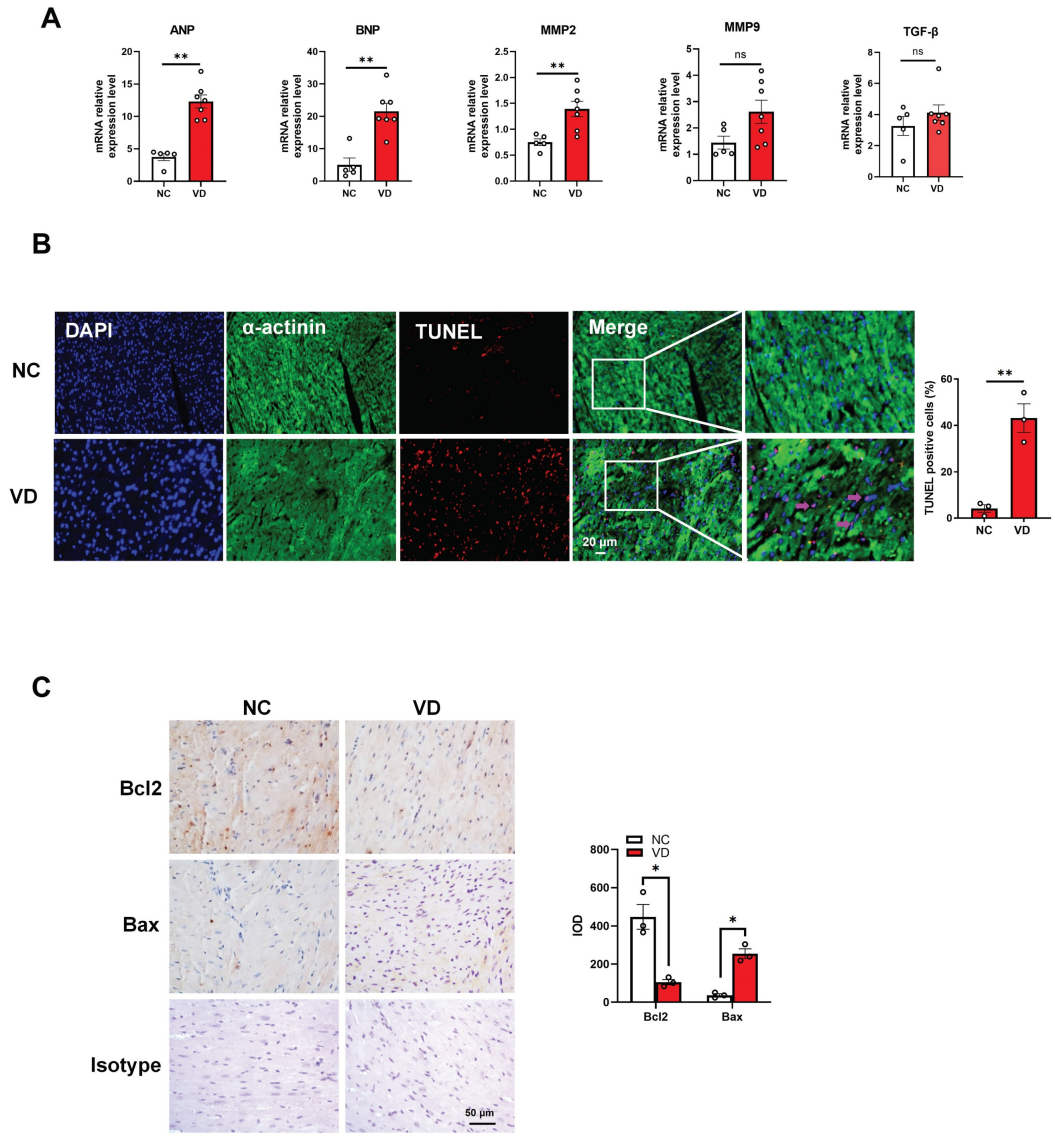
** Genes expression and cardiomyocytes functional changes in hearts from VD_3_-induced VC mice. (A)** mRNA expression levels of ANP, BNP, MMP2, MMP9 and TGF-β in mouse hearts were determined with RT-qPCR analysis (NC = 5, VD = 7). **(B)** Representative images and quantification (n = 3 per group) of TUNEL staining for cardiomyocytes apoptosis in mouse heart tissues. **(C)** Representative images and quantification of immunostaining for protein bcl-2 and bax in mouse heart tissues (n = 3 per group). Data are shown as mean ± SEM. ***P < 0.001. IOD: integrated optical density.

To provide more evidence for cardiac dysfunction in calcified mice, we performed gene expression and cellular analysis using mouse heart samples. The RT-qPCR results indicate that mRNA levels of cardiac dysfunction markers such as ANP and BNP were significantly up-regulated in the VC model compared to the control group. Meanwhile, the mRNA level of MMP2 was significantly increased in VC model, and the same trends were also observed for MMP9 and TGF-β mRNA levels although without significant difference between these two groups (Figure 4A). In response to stress, cardiomyocytes usually undergo apoptosis damage which play critical roles in cardiac dysfunction 25. In this study, TUNEL staining showed remarkably positive apoptotic cardiomyocytes in VC mice model compared with NC group (Figure 4B). Furthermore, immunostaining with the bcl-2 (anti-apoptosis marker) and bax (pro-apoptosis marker) antibodies illustrated the enhanced apoptosis in the heart of VC mice model (Figure 4C). These results suggested the cardiomyocytes apoptosis may responsible for the cardiac dysfunction in VC mice.

### Conditional media from calcified rat VSMCs increases mRNA levels of cardiac genes and apoptosis in rat cardiomyocytes

**Figure 5:**
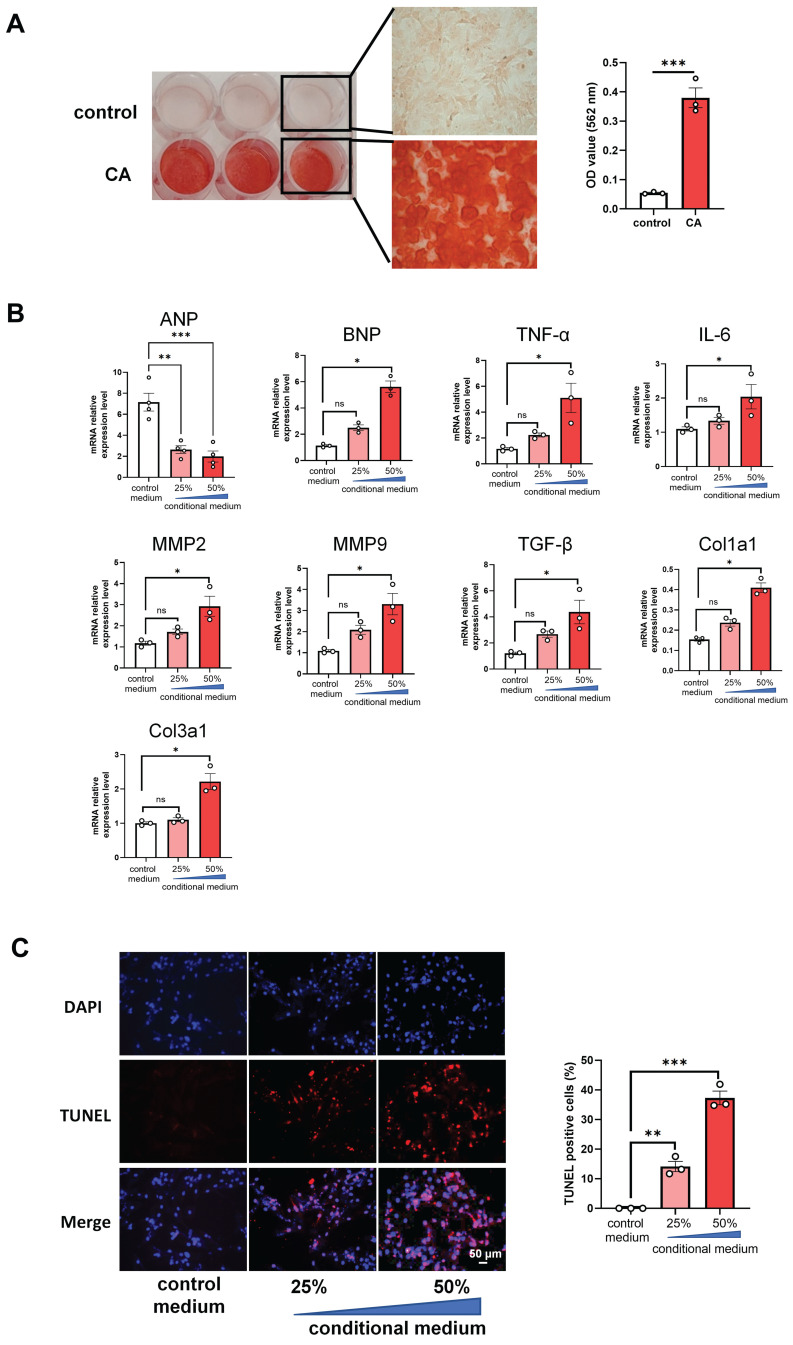
** Conditional media from calcified rat vascular smooth muscle cells (VSMC) induces apoptosis of rat cardiomyocytes. (A)** The primary rat VSMCs were cultured with calcification medium (1.5 mM calcium and 2.0 mM phosphate) for 3 days and then stained with ARS staining. Representative photos of the staining were shown (left), and the OD value after decalcification of ARS staining was quantified (right). **(B)** The collected conditional media from calcified rat VSMCs was used to culture H9C2 cells for 48 h, and mRNA expression levels of ANP, BNP, TNF-α, IL-6, MMP2, MMP9, TGF-β, Col1a1 and Col3a1 in H9C2 cells were determined with RT-qPCR analysis. **(C)** The treated H9C2 cells with conditional media from calcified rat VSMCs were stained with TUNEL. Representative photos and quantification of TUNEL analysis for cardiomyocytes apoptosis. Data from one representative experiment were carried out in triplicate and shown as mean ± SEM. control: cell culture medium; CA: calcification medium. ARS: Alizarin Red S. *P < 0.05, ***P < 0.001.

In order to further uncover whether VC directly affects the apoptosis of cardiomyocytes directly, we cultured H9C2 cells with the conditional media from the calcified RVSMC. As evidenced by ARS staining and its OD value quantification, primary RVSMC was induced remarkable calcification under the calcification medium containing 1.5 mM calcium and 2.0 mM phosphate compared with only DMEM/F12 medium at 48 h - 72 h (Figure 5A). The collected conditional media from the calcified RVSMC at various dilution (25% and 50%) with FBS treated H9C2 cells for further 48 h. Gene expression levels of ANP, BNP, TNF-α, IL-1β, MMP2, MMP9, TGF-β, Col1a1 and Col3a1 were marked increased in the calcified conditional media (50% dilution) treated group compared to the control group (media from only DMEM/F12 treated cells) (Figure 5B). As well, the above calcified conditional media significantly increased the apoptosis of H9C2 cells as shown by TUNEL staining (Figure 5C). Together, these findings indicate that VC has the ability to induce the apoptosis of cardiomyocytes to damage the heart function.

## Discussion

In the present study, we investigated the impact of vascular calcification (VC) in the cardiac function and possible mechanisms involved. Our data provided evidence that C57BL/6 mice developed increased aortic stiffness including elongated vessel length, broken elastic fibers and cardiac dysfunction correlated with severe aortic calcification after chronic VD_3_ stimulation. Furthermore, to the best of our knowledge, we showed for the first time that the apoptosis of cardiomyocytes induced by VC might directly account for the aberrant alteration of cardiac function.

Artery calcification is related to arterial stiffness, and the latter has been suggested as one of the important factors in the development of left ventricular dysfunction through a mechanism termed ventricular-vascular coupling 12. In several animal studies, a positive proportional correlation between aortic calcification and pulse wave velocity (one maker of aortic stiffness) has been noted 26, 27, but only severe calcification, induced by VD_3_ plus nicotine 28 or warfarin plus vitamin K1 in rat models 26, by VD_3_ in obese and insulin-resistant mice (ob/ob) 15 or nephrectomy in uremic mice 27, has been shown to be associated with increased aortic stiffness. We and other groups have previously reported aortic calcification model in rat or mice induced by vitamin D_3_ overdose, accompanied by hypercalcemia, hyperphosphatemia, and increased plasma creatinine, and reduced body weight 17, 29. This model resulted in massive VC, which was observed primarily along the entire aorta in the tunica media, and medial calcification has been reported to be more important than intimal calcium in the development of arterial stiffening and left ventricular changes 30, 31.

In the present study, we found that mice with VD_3_ (350000 IU/kg per day) treatment for 14 consecutive days developed a severe aortic calcification and increased aortic stiffness compared with normal control mice at day 42, as demonstrated by the increase of PPV (a marker of arterial stiffness in mice). Interestingly, we found that PPV values in the 6 weeks of VC were similar to those in the 4 weeks, indicating the severe calcification formation at 4 weeks of VC despite without ARS staining validation in this experiment, which has been evidenced at 4 weeks after VD treatment in our previous study 17. However, we were unable to show the relationship between PPV value and the degree of aortic calcification due to the heavy VC in most of the mice in this model. Moreover, we observed obvious degradation or rupture of the elastic fibers and thickened elastin lamellar in aortae of VD-treated mice, indicating the occurance of abnormal aortic structure in VC mice. Elastolysis has been considered an important niche for VC initiation and progress accompanying arterial stiffness, and modification of aortae's elastic properties decreases vascular recoil and compliance 15 Additionally, taking advantage of the imaging function for vascular structure and blood flow dynamics of LSCI imaging technology, for the first time, we showed that there was a significant decrease of blood flow in carotid artery from VD-treated mice at 6 weeks compared to normal controls, suggesting reduced dilation of arteries in VC mice. The pulsatile hemodynamics in the common carotid artery (CCA) is one of the markers of arterial function, its extent conveys high risk for progression of arterial stiffness to critical cardiovascular diseases 20, 32, 33. Our findings not only support the hypothesis that VC accelerates the aortic stiffness, but also provide a novel hemodynamic assessment for arterial stiffness and risk of cardiac dysfunction in VC mice.

In accordance with the aberrant function alternation of artery, we observed cardiac dysfunction in VD_3_-treated mice at 6 weeks, including decrease of EF, FS, cardiac output, LV mass, heart rate, etc., but these significant alternations in cardiac function showed no obvious difference between 4 (data not shown) and 6 weeks after VD_3_-treated. Considering the mortality of VD-treated mice, we did not extend the experiment time, and employed the mice at 6 weeks for further study. Our cardiac data from this VC mice model were consistent with findings from uremic mice 14, 27 or rats with nephrectomy 34 providing more evidence for the critical role of VC in the development of cardiac disorders. However, the role of cardiomyocytes contributing to cardiac impairment induced by VD has so far not been documented. Here we observed that cardiomyocytes apoptosis was involved. TUNEL staining results revealed a significant increase of apoptotic cardiomyocytes (labelled with α-actinin) in the heart of VD_3_-treated mice. In parallel, anti-apoptosis protein bcl-2 was clearly highly expressed in normal mice, and pro-apoptosis protein bax was inversely abundantly expressed in the heart of VD_3_-treated mice. Overall, our data from this VC mice model suggest VC accelerated cardiac impairment partially via cardiomyocytes apoptosis. In a similar study, Amann et al. demonstrated cardiomyocyte apoptosis and hypertrophy contributed to left ventricular dysfunction in a short-term moderate experimental renal failure rat model by nephrectomy 35, and this finding strongly supports our hypothesis that VC leads to cardiac damage via cardiomyocytes apoptosis.

Accumulating evidence suggest that VSMC calcification plays a central role in the development and progression of VC, and primary rat VSMC calcification has been successfully induced *in vitro* under the calcification medium (1.5 mM calcium and 2.0 mM phosphate) in our and other group studies 17, 21. We here further investigated the direct effect of VC on rat cardiomyocytes H9C2 *in vitro* with VC conditional media collected from rat VSMC calcification model. Various dilution of VC conditional media induced significant increase mRNA levels of cardiac genes ANP and BNP, inflammatory cytokines TNF-α, IL-6, as well as remodeling and fibrosis genes MMP2, MMP9, TGF-β, Col1a1 and Col3a1 in H9C2 cells. Meanwhile, consistent with our finding of cardiac apoptosis in VD_3_-treated mice, rat cardiomyocytes H9C2 displayed marked apoptosis when treated with conditional rat VSMC calcification media. Our results confirmed the direct effect of cardiomyocytes apoptosis induced by VC resulting in cardiac dysfunction. Besides, we also found plasma from CAD or CKD patient with CAC increased mRNA levels of ANP, BNP and TGF-β in H9C2 cells as well as the apoptosis of H9C2 cells with a concentration dependent manner (Supplementary Figure 2), indirectly indicating the potential relationship of cardiomyocytes apoptosis and VC presence whatever in CAD or CKD patients. Several studies have identified various factors released from VSMCs in calcification condition, including inflammation, oxidative stress, MMP activity, apoptosis and autophagy, etc 36. Recently, the discovery of extracellular vesicles secreted during the VC process emphasizes the importance of intercellular/interorgan crosstalks and suggests potentially novel preventive or therapeutic strategies for VC-related diseases 37. Nevertheless, the specific factors produced in VC contributing to cardiomyocytes apoptosis remain to be determined in future studies. To our knowledge, this is the first study demonstrating this phenomenon in the process of ventricular-vascular coupling in VD-induced calcification.

The present study has several limitations. First, we cannot analyze the time course of changes in cardiac and aortic function parameters and the close association with aortic calcification quantification in this VC mice model. In this study, we examined the calcification of mouse aorta at the end of the experiment due to lacking desired VC detection technology in mice. Thus, we conducted *in vitro* study to provide evidence for the association with aortic VC and cardiac dysfunction. Second, we cannot exclude the possible contribution of kidney damage to cardiac dysfunction because VD overdose is toxic to kidney. In fact, we found kidney impairment and calcification in this mice model (Supplementary Figure 1) as that in uremic mice with nephrectomy. Third, the significant loss of body weight in this disease model might interfere with the global hemodynamic, nutritional and biochemical processes, thus potentially confounding some of our findings.

## Conclusions

We found that mice after chronic VD_3_ stimulation developed moderate or severe aortic calcification, increased aortic stiffness and impaired cardiac function compared with non-treated mice. The apoptosis of cardiomyocytes induced by VC might directly account for the aberrant alteration of cardiac function. These new evidence for the role of VC underlying cardiac dysfunction provide a new therapeutic strategy for VC-related cardiovascular diseases.

## Supplementary Material

Supplementary methods, figures and tables.

## Abbreviations

VC: vascular calcification
VSMC: vascular smooth muscle cells
CAC: coronary artery calcification
CAD: coronary artery disease
CKD: chronic kidney disease
