# Supplementary material for: Aortic calcification accelerates cardiac dysfunction via inducing apoptosis of cardiomyocytes
Source: Int J Med Sci. 2024 Jan 1;21(2):306–18. doi: 10.7150/ijms.90324 (PMC10758138; doi:10.7150/ijms.90324)
Supplement: Supplementary file 1 — Supplementary methods, figures and tables. [file ijmsv21p0306s1.pdf]

SUPPLEMENTARY MATERIALS

Aortic calcification accelerates cardiac dysfunction via inducing apoptosis of cardiomyocytes

Nannan Hao[1, *], Hui Yong[1, *], Feifei Zhang[1, *], Chang Liu[2], Yulu Qiu[2], Yumeng Shi[2], Chunjian Li[1, ǂ], Fang Wang[1, ǂ]

[1]Department of Cardiology, the First Affiliated Hospital of Nanjing Medical University, China

[2]Department of Rheumatology, the First Affiliated Hospital of Nanjing Medical University, China

*These authors contributed equally to this work.

[ǂ]Correspondence to: Dr. Fang Wang, E-mail: wangfangheart@njmu.edu.cn, or Chunjian Li, lijay@njmu.edu.cn, Department of Cardiology, the First Affiliated Hospital of Nanjing Medical University, Nanjing 210029, China.

Supplementary methods

Patient blood samples

Blood samples were collected from peripheral veins and centrifuged to obtain plasma from 3 CAD patients (aged 67 ± 2.65 years, 2 males and 1 female), 3 CKD patients (aged 54 ± 4.51 years, 2 males and 1 female) and 3 healthy controls (aged 36 ± 3.71 years, 2 males and 1 female). All patients underwent coronary computed tomography angiogram (CTA) with coronary artery calcification (Agatston score > 400). This study was approved by the ethics committee of the First Affiliated Hospital of Nanjing Medical University.

Plasma from patients treated H9C2 cells

To study effect of peripheral blood from patients with vascular calcification (VC) on cardiomyocytes function, we cultured H9C2 cells with plasma collected from both CAD and CKD patients with coronary artery calcification as well as healthy controls at different dilutions with FBS. After 48 h, H9C2 cells were collected for RT-qPCR analysis (ANP, BNP and TGF-β) and TUNEL staining, respectively.

Von Kossa staining

Von Kossa staining was used for calcification determination. The paraffin-embedded heart and kidney from mice were stained with a von Kossa staining kit according to the manufacturer’s protocol. Briefly, after rinsing in ddH2O, sections were incubated with 1% silver nitrate solution under a 60 watt light bulb for 1 hour. After washing, 5%

sodium thiosulfate was added to the slides for 5 min to remove un-reacted silver. Calcified areas are shown as black or brown black deposits.

Supplementary figures

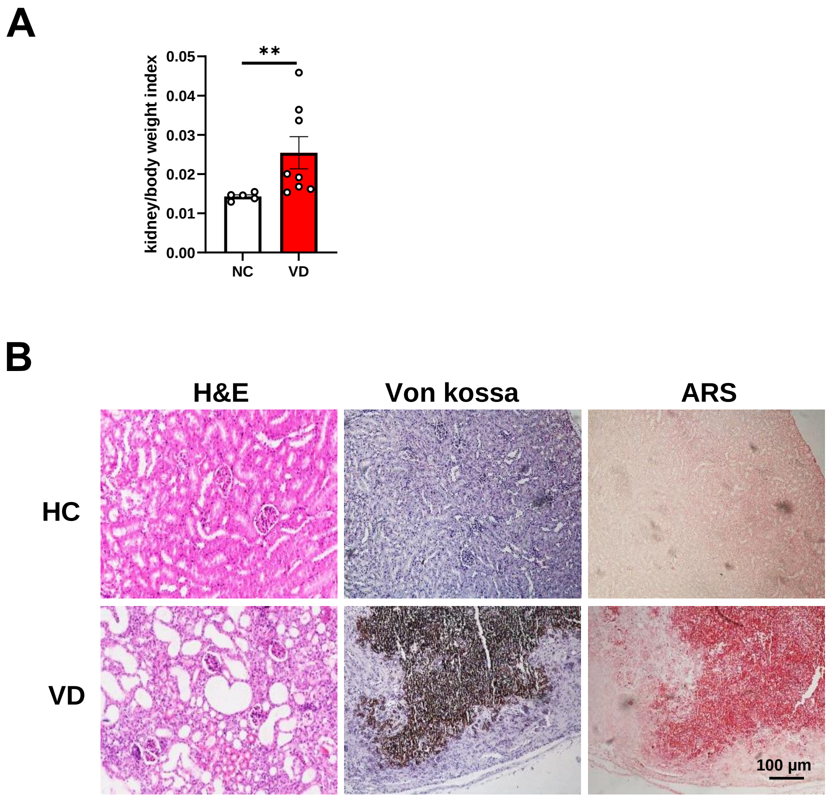

Supplementary Figure 1. The kidney changes of VD_3-induced VC mice. (A) C57BL/6 mice were intraperitoneally injected with VD_3 (350,000 IU/kg/day) for 14 days. At day 42, kidney samples were collected and the ratio of kidney to body weight was determined. Values are shown as mean ± SEM (NC = 5, VD = 8). $^{**}P < 0.01$. **(B)** Representative images of H&E, Von kossa and ARS staining for mice kidney, respectively. VC: vascular calcification; ARS: Alizarin Red S.

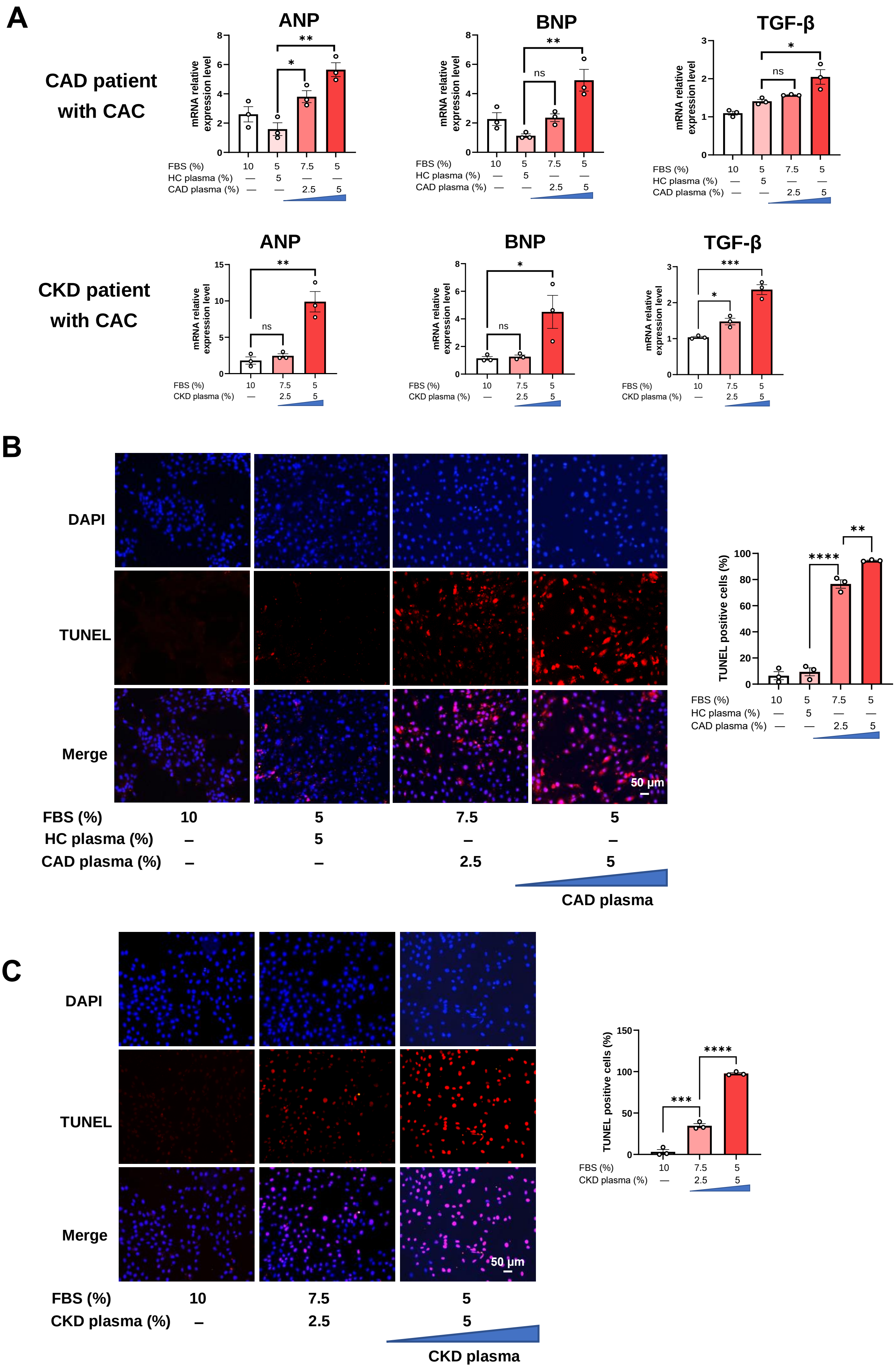
A
CAD patient with CAC
ANP
BNP
TGF-β
mRNA relative expression level
FBS (%) 10 5 7.5 5
HC plasma (%) — 5 — —
CAD plasma (%) — — 2.5 5
ns
CKD patient with CAC
FBS (%) 10 7.5 5
CKD plasma (%) — 2.5 5
B
DAPI
TUNEL
Merge
50 μm
FBS (%) 10 5 7.5 5
HC plasma (%) – 5 – –
CAD plasma (%) – – 2.5 5
CAD plasma
TUNEL positive cells (%)
C
DAPI
TUNEL
Merge
50 μm
FBS (%) 10 7.5 5
CKD plasma (%) – 2.5 5
CKD plasma
TUNEL positive cells (%)

Supplementary Figure 2. Plasma from CAD or CKD patients induces apoptosis of rat cardiomyocytes. (A) RT-qPCR analysis of the expression of cardiac dysfunction related genes (ANP, BNP and TGF-β) in H9C2 cells under different plasma from patients with CAD, CKD and healthy controls (HC) for 48 h. **(B-C)** Representative images and quantification of TUNEL staining for H9C2 cells apoptosis with different plasma from CAD patients and healthy controls (HC) **(B)** as well as CKD patients **(C)** for 48 h. Data from one representative experiment carried out in triplicate and shown as mean ± SEM. $*P < 0.05$, $**P < 0.05$, $***P < 0.001$. CAD: coronary artery disease; CKD: chronic kidney disease; CAC: coronary artery calcification.

Supplementary tables

Table 1 Primers used for quantitative real time PCR

Gene symbol	Forward (5'-3')	Reverse (5'-3')
mouse ANP	AGGCAGTCGATTCTGCTT	CGTGATAGATGAAGGCAGGAAG
mouse BNP	TAGCCAGTCTCCAGAGCAATTC	TTGGTCCTTCAAGAGCTGTCTC
mouse MMP2	CAAGTTCCCCGGCGATGTC	TTCTGGTCAAGGTCACCTGTC
mouse MMP9	GACGACATAGACGGCATCC	TGGTTCAGTTGTGGTGGTG
mouse TGF-β	CTCCCGTGGCTTCTAGTGC	GCCTTAGTTTGGACAGGATCTG
mouse β-actin	CCTCACTGTCCACCTTCC	GGGTGTAAAACGCAGCTC
rat ANP	GCCGGTAGAAGATGAGGTCA	GGGCTCCAATCCTGTCAATC
rat BNP	ATCTGTCGCCGCTGGGAGGT	TGGATCCGGAAGGCGCTGTC
rat TNFα	CAGCCAGGAGGGAGAAC	GTATGAGAGGGACGGAACC
rat IL-6	CCGTTTCTACCTGGAGTTTGT	GTTTGCCGAGTAGACCTCATAG
rat MMP2	TTGACCAGAACACCATCG	CTTGCGGGGAAAGAAGT
rat MMP9	CGCTGGGCTTAGATCATT	TGCTGGATGCCTTTTATGT
rat TGF-β	CCTACATTTGGAGCCTGGA	CCGGGTTGTGTTGGTTG
rat Col1a1	GAGCGGAGAGTACTGGATCGA	CTGACCTGTCTCCATGTTGCA
rat Col3a1	TGCCATTGCTGGAGTTGGA	GAAGACATGATCTCCTCAGTGTTGA
rat β-actin	CCCGCGAGTACAACCTTCT	CGTCATCCATGGCGAACT

Table 2 Body and organ weights of normal and VD_3-induced VC mice

**Indicators**	**NC group (n = 5)**	**VD group (n = 8)**
Body weight (g)	25.03 ± 0.55	15.21 ± 0.43**
Heart weight (g)	0.12 ± 0.002	0.08 ± 0.002**
Liver weight (g)	1.24 ± 0.069	0.78 ± 0.06**
kidney weight (g)	0.36 ± 0.009	0.39 ± 0.07

Data are presented as mean ± SEM. NC: normal control; VD: VD_3-treated mice. $^{**}P < 0.01$.

Table 3 Echocardiographic data of left ventricle in normal and VD_3-induced VC mice

**Indicators**	**NC (n = 5)**	**VD (n = 10)**	**P value**
FS (%)	45.830 ± 1.711	41.380 ± 1.035	0.035*
LVEF (%)	77.620± 1.614	72.950 ± 1.064	0.044*
LVMI (g/m2)	15.710 ± 0.483	14.850 ± 0.403	0.218
Heart rate	595.00 ± 19.65	445.30 ± 19.24	0.0003***
LV Mass Cor	100.400 ± 3.327	71.90 ± 5.24	0.003*
Volume; d (μl)	50.720 ± 2.371	44.130 ± 1.908	0.059
Diameter; d (mm)	3.491 ± 0.067	3.292 ± 0.059	0.063
Diameter; s (mm)	1.895 ± 0.092	1.930 ± 0.051	0.723
LVAWT; s (mm)	1.639 ± 0.037	1.381 ± 0.049	0.005**
LVAWT; d (mm)	1.040 ± 0.033	0.909 ± 0.028	0.014*
LVPWT; d (mm)	0.916 ± 0.010	0.817 ± 0.018	0.003**
LVPWT; s (mm)	1.411 ± 0.016	1.245 ± 0.023	0.0004***
Stroke Volume (μl)	39.390± 1.183	32.340± 1.377	0.006**
Cardiac Output (mL/min)	23.420 ± 0.895	14.310 ± 0.643	< 0.0001***
PWTF (%)	58.030 ± 3.832	51.800± 2.227	0.156
CI	2949.0 ± 181.2	2166.0 ± 119.0	0.003**

Data are presented as mean ± SEM. *P < 0.05, **P < 0.01, ***P < 0.001. FS: fractional shortening; LVEF: left ventricular ejection fraction; LVMI: left ventricular mass index; d: diastolic diameter; s: systolic diameter; LVAWT: left ventricular anterolateral wall thickness; LVPWT: left ventricular posterior wall thickness; PWTF: posterior wall thickening rate; CI: cardiac index.
